# Fecopneumothorax and colopleural fistula – uncommon complications of Crohn's disease

**DOI:** 10.1186/1471-230X-6-17

**Published:** 2006-06-06

**Authors:** G Barišiæ, Z Krivokapiæ, T Adžiæ, A Pavloviæ, M Popoviæ, M Gojniæ

**Affiliations:** 1 Institute for Digestive Diseases, Clinical Center of Serbia, Belgrade, Serbia and Montenegro; 2 Institute for Lung Diseases and Tuberculosis, Clinical Center of Serbia, Belgrade, Serbia and Montenegro; 3 Institute of Gynecology and Obstetrics, Clinical Center of Serbia, Belgrade, Serbia and Montenegro

## Abstract

**Background:**

Colopleural fistula and fecopneumothorax are very rare complications of Crohn's disease. Fistula formation is frequent in Crohn's disease and occurs in approximately 33% of patients. On the other hand, fistulous communication between the pleural cavity and adjacent organs below the diaphragm is extremely rare.

**Case presentation:**

We describe the case of 27 year-old female with colopleural fistula as a complication of Crohn's disease. The diagnosis was established with clinical exam, barium enema, chest X-ray, abdominal and chest CT exam. The treatment was surgical.

**Conclusion:**

Colopleural fistula and fecopneumothorax are rare but life treating complications of Crohn's disease. Surgical treatment is mandatory as soon as the diagnosis is established.

## Background

Fistulous communication between the colon and adjacent structures below the diaphragm is known but rare complication of Crohn's disease. We present a case of 27 years-old patient suffering Crohn's disease with fecopneumothorax and colopleural fistula as complications of the disease.

## Case presentation

Young female, 27 years-old, was admitted as an emergency in December 2002 at the Institute for Lung Diseases. Her general condition was poor revealing severely ill patient. She was febrile up to 39°C, dyspnoic, exhausted, with strenuous dry cough, with Body Mass Index 12, with hypoproteine pitting edema of lower extremities. Respiratory sounds were diminished on the left side. Physical exam of the abdomen revealed rigidity and guarding at the left hypochondrial quadrant. Abdominal sounds were diminished. Laboratory findings at presentation were: Hemoglobin 85 g/L; Leukocytes 24,2 × 10^9^/L; Platelets 110 × 10^9^/L; Albumin 26 g/L; Sed. rate 76/h. She suffered from Crohn's colitis since 1999 when the diagnosis was established and was occasionally on peroral therapy with Budesonid and Salofalk combined with Salofalk enemas. She experienced three severe attacks during the course of the disease which required hospitalization and aggressive medical treatment prior the operation. Colonoscopy performed one year prior the operation revealed Crohn's colitis from anal canal to the level of transverse colon, with serpiginous ulcerations and narrowed lumen.

**Figure 1:**
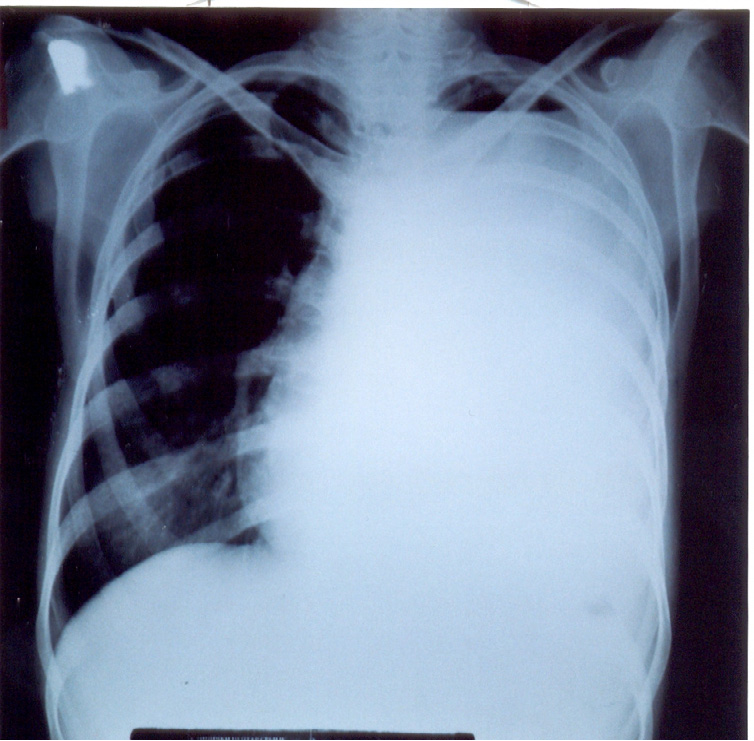
Chest X-Ray reveals hydropneumothorax on the left side.

Chest radiography showed hydropneumothorax on the left side (figure 1). Thoracocentesis was immediately performed and revealed feculent effusion. It was followed with the chest tube insertion in the left pleural space. Total of 3500 ml of feculent smelling fluid was drained out of the left pleural space and sent for bacterial culture which revealed mixed infection with Staphylococcus, Enterococcus, Klebsiella, Escherichia coli and Pseudomonas.

Abdominal ultrasound showed hypoechoic tube-like structure from the lower pole of the spleen to the left pleural space.

**Figure 2:**
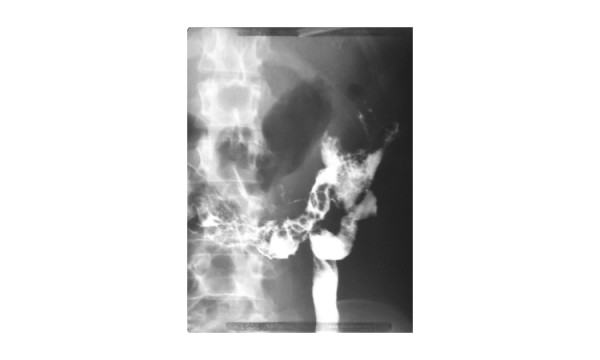
Spreading of contrast material at splenic flexure region, both proximally and supradiaphragmatically.

Barium enema showed narrowed colon with lacunar defects and extensive fistulous tracks at the splenic flexure with two of them spreading towards the left diaphragm. Later serial shots clearly revealed that barium enema reaches the left pleural space (Fig. 2).

Consultant colorectal surgeon was requested and he transferred the patient to the colorectal department. Following resuscitation and short preoperative preparation the patient was operated. After midline laparotomy and thorough exam of abdominal cavity, a rigid mass, 15 cm in diameter was found adherent to the colon, tail of the pancreas, spleen and a part of the left diaphragm. Other parts of the colon were also altered with obvious signs of acute inflammation including cecum and ascending colon. Terminal ileum and colon were dilated up to the level of the splenic flexure which was involved into the inflammatory mass. The rest of the colon below splenic flexyre was not dilated but was also macroscopically altered, appearing as Crohn's colitis. Rectum was affected too, but inflammation was not so severe as in other parts of the colon. After mobilization of splenic flexure, two fistulous tracts with granulation tissue were encountered. One was arising from the splenic flexure of colon and was directed towards the retroperitoneal space beyond the left kidney. The second fistulous tract was directed upwards, between the colon, tail of the pancreas and spleen towards the left diaphragm. After splenectomy and curettage of the fistulous tract, an opening measuring 1 cm in diameter was found in the left diaphragm connected to the pleural cavity. Total colectomy with terminal Brooke ileostomy was performed as one as splenectomy, suture of the left diaphragm and chest tube drainage of the left pleural space. Rectum was transected at the level of promontory and sutured in two layers with interrupted absorbable sutures.

**Figure 3:**
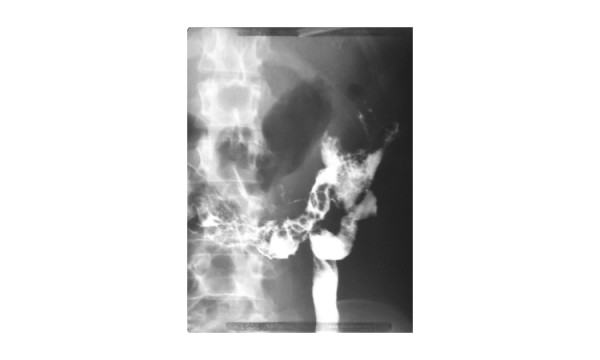
The specimen-severe form of Crohn's colitis with forceps pointing fistulous opening.

At fourth postoperative day digestive functions recovered, abdominal drains were removed and patient was referred to the Institute for Lung Diseases for further treatment. Pathological exam of the resected specimen revealed severe form of Crohn's colitis with transmural inflammation and thickening of the bowel wall. Inflammatory changes were most prominent in the region of the fistulous opening where the wall of colon was thickened with narrowed lumen and strictures (figure 3) Right colon also harbored the signs of inflammation but was relatively spared compared to the transverse and left colon.

The patient was discharged from hospital on 32^nd ^postoperative day in good condition with normal chest radiography. No further specific therapy addressed to Crohn's disease was warranted. At the last outpatient visit one year ago, she was doing well without any medicatons and improved BMI (25.3).

## Discussion

To the best of our knowledge, this is the first reported case of fecopneumothorax and colopleural fistula complicating Crohn's disease.

Fistula formation is frequent in Crohn's disease and occurs in approximately 33% of patients [1]. On the other hand, fistulous communication between the pleural cavity and adjacent organs below the diaphragm is extremely rare complication of Crohn's disease. The pathophysiology of fistulous tracts development in Crohn's disease is yet unknown. A number of case reports regarding fistulas in Crohn's disease were published including pancreaticopleural, gastropleural, duodenopleural, colobronchial, cholecystopleural, bronchobiliar [2] and bronchopleurobiliar. Colobronchial fistulas have been quite frequently described in Crohn's disease, in most cases between splenic flexure of colon and left bronchial tree [3-5] and in one case between splenic flexure of colon, stomach and left bronchial tree [6].

Recurrent pneumonia with feculent sputum in patients with Crohn's disease should raise suspicion of colobronchial fistula. Diagnosis of fecopneumothorax is based on meticulous clinical exam and additional diagnostic procedures. The patient is in an acute respiratory distress, tachycardic, tachypnoic and hypotensive. Respiratory sounds are weakened or silent on the diseased side.

Feculent discharge from the chest tube should raise suspicion of colopleural fistula and should require immediate chest X-ray along with barium enema. Abdominal and thoracic CT scan or MRI could provide additional information about the stage of the disease and can exclude the presence of abscess or fluid collection in abdominal cavity.

In presented case, communication between the splenic flexure of colon and left pleural space was clearly revealed with barium enema. Partial collapse of the left lung persisted after surery, and required resides of chest tube up to 31 days. Full expansion of the left lung after intensive physical rehabilitation occurred thus making thoracotomy and decortication of the left lung unnecessary.

## Conclusion

Fecopneumothorax and colopleural fistula are life-threating but extremely rare complication of Crohn's disease. Urgent surgical treatment is necessary as soon as the diagnosis is established based on clinical and radiological findings.

## Competing interests

The author(s) declare that they have no competing interests.

## Authors' contributions

GB designed the study and drafted the manuscript

ZK was consultant surgeon who operated the patient

TA patcicipated in establishing diagnosis and medical treatment

AP participated in medical treatment

MP participated in the design of the stidy

MG participated in the design of the study

All authors read and approved the final manuscript

## Pre-publication history

The pre-publication history for this paper can be accessed here:
